# A review of the genus *Toxorhina* Loew from China, with descriptions of three new species (Diptera, Limoniidae, Limoniinae)

**DOI:** 10.3897/zookeys.480.7526

**Published:** 2015-02-02

**Authors:** Xiao Zhang, Yan Li, Ding Yang

**Affiliations:** 1 Department of Entomology, China Agricultural University, Beijing 100193, China; 2 Plant Protection College, Shenyang Agricultural University, Shenyang 110866, China

**Keywords:** China, Diptera, Limoniidae, new species, *Toxorhina*

## Abstract

The genus *Toxorhina* Loew from China is reviewed. Seven species belonging to the subgenus *Ceratocheilus* Wesche are recognized, of which three species, Toxorhina (Ceratocheilus) huanglica **sp. n.**, Toxorhina (Ceratocheilus) omnifusca **sp. n.** and Toxorhina (Ceratocheilus) univirgata **sp. n.**, are described as new to science, Toxorhina (Ceratocheilus) fuscolimbata Alexander is recorded from China for the first time, and three known species are redescribed and illustrated.

## Introduction

**Figure 1.**
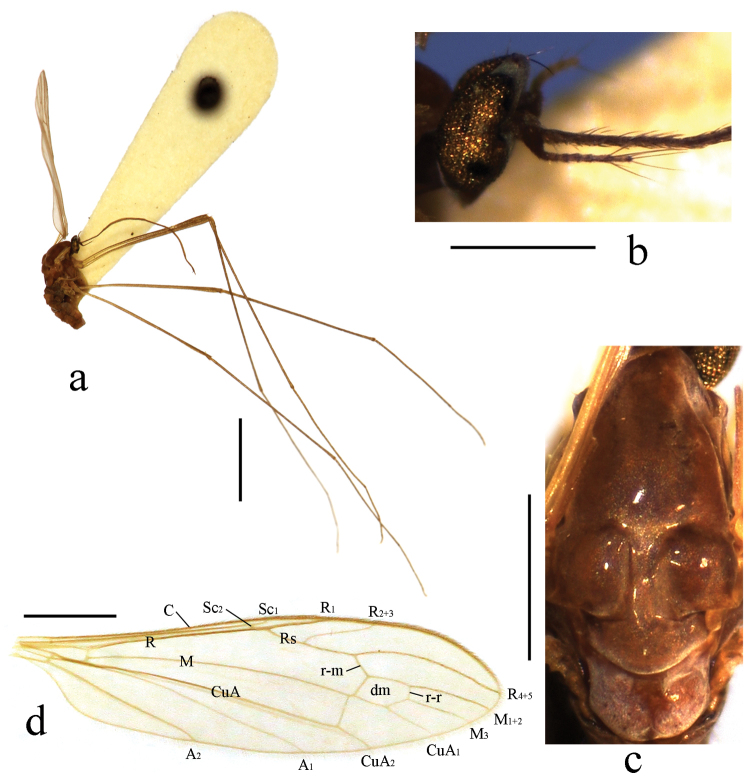
Toxorhina (Ceratocheilus) formosensis Alexander, 1928. Holotype. **a** Male habitus, lateral view **b** Head, lateral view **c** Thorax, dorsal view **d** Wing. Scale bar: **a** = 2.0 mm; **b** = 0.5 mm; **c** = 0.5 mm; **d** = 1.0 mm.

The genus *Toxorhina* was erected by Loew in 1850. Its detailed features for the recognition are given by Osten Sacken (1869) and Alexander (1947). The genus is well defined by the elongate rostrum which is longer than head and thorax combined together. The genera *Elephantomyia* Osten Sacken and *Helius* Lepeletier & Serville have same feature (mouth parts of the genus *Geranomyia* Haliday are strongly elongate, but rostrum short (Podenas and Gelhaus 2007)), but *Toxorhina* can be easily distinguished from them by the antenna with 12 or less segments and with long hairs on outer two segments only (Fig. 1: d), the wing with much reduced venation, and the legs with profoundly bifid hairs (Fig. 3: a). The genus *Toxorhina* is divided into three subgenera: *Ceratocheilus* Wesche, 1910, *Toxorhina* (s. str.) and *Eutoxorhina* Alexander, 1934. The subgenus *Ceratocheilus* is easily distinguished from the other two subgenera by the wing with two branches of Rs reaching the margin (Fig. 1: d). In the subgenera *Toxorhina* and *Eutoxorhina*, the wing has a single branch of Rs reaching the margin.

The genus *Toxorhina* is widely distributed in the world except the Palaearctic Region. It has 148 known species, of which 2 species are from the Nearctic Region, 37 species are from the Neotropic Region, 36 species are from the Afrotropical Region, 33 species are from the Oriental Region, and 40 species are from the Australasian (Oceanian) Region. The subgenus *Ceratocheilus* has 77 known species, of which 12 species are from the Neotropic Region, 24 species are from the Afrotropical Region, 17 species are from the Oriental Region, and 24 species are from the Australasian (Oceanian) Region (Oosterbroek 2014).

This paper redescribed and illustrated three known species and one new record from China, including detailed descriptions and illustrations of male of Toxorhina (Ceratocheilus) tinctipennis, Toxorhina (Ceratocheilus) taiwanicola for the first time. In addition, three new species, Toxorhina (Ceratocheilus) huanglica sp. n., Toxorhina (Ceratocheilus) omnifusca sp. n. and Toxorhina (Ceratocheilus) univirgata sp. n., are described and illustrated.

## Material and methods

The specimens were studied and illustrated with ZEISS Stemi 2000-c stereo microscope. Details of coloration were mainly checked in specimens immersed in 75% ethyl alcohol (C_2_H_5_OH). Genitalic preparations were made by macerating the apical portion of the abdomen in cold 10% NaOH for 12–15 hours. After examination it was transferred to fresh glycerin (C_3_H_8_O_3_) and stored in a micro vial pinned below the specimen. Type specimens of Toxorhina (Ceratocheilus) formosensis, Toxorhina (Ceratocheilus) taiwanicola and Toxorhina (Ceratocheilus) tinctipennis are deposited in USNM (the National Museum of Natural History, Smithsonian Institution, Washington D.C., USA). The other studied specimens, including type specimens of Toxorhina (Ceratocheilus) huanglica sp. n., Toxorhina (Ceratocheilus) omnifusca sp. n. and Toxorhina (Ceratocheilus) univirgata sp. n., are deposited in CAU (the Entomological Museum of China Agricultural University, Beijing, China).

The morphological terminology mainly follows McAlpine (1981), and the venation is described after Alexander and Byers (1981). Terminology of male hypopygium changes according to Ribeiro (2006): lobe of gonostylus = inner gonostylus, clasper of gonostylus = outer gonostylus. The following abbreviations in figures are used: tg 9/10 = tergite nine/ten, st 9 = sternite nine, goncx = gonocoxite, gonst = gonostylus, c gonst = clasper of gonostylus, l gonst = lobe of gonostylus, aed = aedeagus, interb = interbase, cerc = cercus, hyp vlv = hypogynial valve.

## Taxonomy

### Key to species of subgenus *Ceratocheilus* from Oriental Region

**Table:** 

1	Wing with darkened seams along several veins (Fig. 3d)	**2**
–	Wing unpatterned	**4**
2	Wing with basal section of CuA_1_ beyond fork of M (Fig. 3d)	***Toxorhina fuscolimbata* Alexander, 1967** (**China**; India)
–	Wing with basal section of CuA_1_ at or close to fork of M	**3**
3	Size medium (wing length 5.0 mm); legs with coxae and trochanters yellow, fore pair weakly darkened; haltere pale yellow (Alexander 1956)	***Toxorhina capnitis* Alexander, 1956** (Thailand)
–	Size relatively large (wing length 6.5 mm), legs with coxae ochreous, trochanters blackish; haltere white (Edwards 1926)	***Toxorhina majus* (Edwards, 1926)** (Malaysia)
4	Pleuron with conspicuous darkened stripe(s) (Figs 3a, 5a, 13a)	**5**
–	Pleuron uniformly concolorous or with darker or paler region, without conspicuous stripe	**8**
5	Wing with R_2+3_ ending before end of basal section of R_4+5_, R_2+3_ nearly perpendicular at origin (Alexander 1929)	***Toxorhina romblonensis* (Alexander, 1929)** (Philippines)
–	Wing with R_2+3_ ending at or beyond end of basal section of R_4+5_, R_2+3_ not as above	**6**
6	Abdomen bicolored (Fig. 5a); gonocoxite of male hypopygium with a blunt lobe near base (Fig. 6a–b)	***Toxorhina huanglica* sp. n. (China)**
–	Abdomen not as above; gonocoxite of male hypopygium without lobe near base	**7**
7	Male hypopygium with rods of aedeagus short (Alexander 1962, 1970)	***Toxorhina monostyla* Alexander, 1962** (India)
–	Male hypopygium with rods of aedeagus long (Fig. 14a–b)	***Toxorhina univirgata* sp. n.** (**China**)
8	Wing with basal section of CuA_1_ beyond fork of M	**9**
–	Wing with basal section of CuA_1_ before or close to fork of M	**10**
9	Antenna black throughout; rostrum shorter than wing or remainder of body (Alexander 1967; Alexander 1970)	***Toxorhina bistyla* Alexander, 1967** (India)
–	Antenna black with scape obscure yellow; rostrum longer than wing or remainder of body (Brunetti 1918; Alexander 1936; Joseph 1979)	***Toxorhina brevifrons* (Brunetti, 1918)** (India)
10	Abdomen light brown, hinder half of segments distinctly darker	***Ceratocheilus latifrons* (Brunetti, 1918)** (Malaysia)
–	Abdomen not as above	**11**
11	Male hypopygium with rods of aedeagus long	**12**
–	Male hypopygium with rods of aedeagus short	**14**
12	Rostrum longer than wing (Alexander 1967)	***Ceratocheilus fulvicolor* Alexander, 1967** (India)
–	Rostrum shorter than wing	13
13	Wing with R_2+3_ short, with a rather strong double curve; male hypopygium with gonostylus rather slender and mainly pale, with a long black tooth in middle at right angles to main axis (Edwards 1933)	***Ceratocheilus contractifrons* (Edwards, 1933)** (Malaysia)
–	Wing with R_2+3_ long, gently sinuous (Fig. 1d); male hypopygium with gonostylus stout and entirely blackened, outer lateral angle with a slender rod (Fig. 2)	***Ceratocheilus formosensis* (Alexander, 1928) (China)**
14	Gonocoxite of male hypopygium without lobe near base	**15**
–	Gonocoxite of male hypopygium with a blunt lobe near base	**16**
15	Wing with Sc_1_ ending near middle of Rs; gonocoxite of male hypopygium with two dense areas of setae beyond middle and near apex (Alexander 1962; Alexander 1970)	***Ceratocheilus luteibasis* Alexander, 1962** (India)
–	Wing with Sc_1_ just before origin of Rs (Fig. 7d); gonocoxite of male hypopygium not as above	***Ceratocheilus omnifusca* sp. n. (China)**
16	Pleuron uniformly brownish black or black	**17**
–	Pleuron dark brown with ventral region paler	**18**
17	Wing with a strong brown suffusion, R_2+3_ ending beyond or close to end of basal section of R_4+5_ (Fig. 11e)	***Ceratocheilus tinctipennis* (Alexander, 1930)** (**China**)
–	Wing subhyaline with base more yellowed, R_2+3_ ending before end of basal section of R_4+5_ (Alexander 1966)	***Ceratocheilus tuberifera* Alexander, 1966** (India)
18	Male hypopygium with gonostylus as long as gonocoxite (Alexander 1967)	***Ceratocheilus simplicistyla* Alexander, 1967** (India)
–	Male hypopygium with gonostylus conspicuously shorter than gonocoxite	**19**
19	Wing with R_2+3_ ending beyond end of basal section of R_4+5_; gonostylus of male hypopygium with a spine near basal third (Alexander 1936; Alexander 1970)	***Ceratocheilus mesorhyncha* Alexander, 1936** (India)
–	Wing with R_2+3_ ending before end of basal section of R_4+5_ (Fig. 9e); gonostylus of male hypopygium with a spine near middle (Fig. 10a–b)	***Ceratocheilus taiwanicola* (Alexander, 1923)** (**China**)

#### Toxorhina (Ceratocheilus) formosensis

Taxon classificationAnimaliaDipteraLimoniidae

Alexander, 1928

Figs 1 , 2

Ceratocheilus formosensis Alexander 1928: 479. Type locality: Mt. Rantaizan, China (Taiwan).

##### Diagnosis.

Rostrum shorter than wing. Prescutum brown with three broad dark brown stripes. Pleuron generally dark brown. Wing tinged pale grey; R_2+3_ ending beyond end of basal section of R_4+5_, basal section of CuA_1_ before fork of M. Abdomen Generally brown to dark brown. Gonostylus stout and entirely blackened, outer lateral angle with a slender rod. Rods of aedeagus long.

##### Description.

Male. Body length 4.2 mm, wing length 5.2 mm, rostrum length 4.0 mm.

Head (Fig. 1b). Brownish black. Hairs on head black. Antenna length 0.4–0.5 mm, black. Pedicel enlarged and nearly globose. First flagellomere subconical; remaining flagellomeres cylindrical, each flagellomere longer and slenderer than previous one, terminal two flagellomeres longest with several long hairs. Rostrum black with black hairs.

Thorax. Generally brown to dark brown. Pronotum brown. Prescutum brown with three broad dark brown stripes. Scutum dark brown with middle area paler. Scutellum and mediotergite brown (Fig. 1c). Pleuron (Fig. 1a) generally dark brown. Hairs on thorax dark brown. Coxae and trochanters brownish yellow; femora dark brown with bases paler; tibiae and tarsi dark brown. Wing (Fig. 1d) tinged pale grey; veins pale brown. Venation: Sc_1_ ending nearly at origin of Rs, Sc_2_ near its tip; R_2+3_ ending beyond end of basal section of R_4+5_; basal section of CuA_1_ before fork of M; A_1_ curved relatively smooth. Haltere length 0.7 mm, pale brownish yellow with knob darker.

Abdomen (Fig. 1a). Generally brown to dark brown. Hairs on abdomen dark brown.

**Figure 2.**
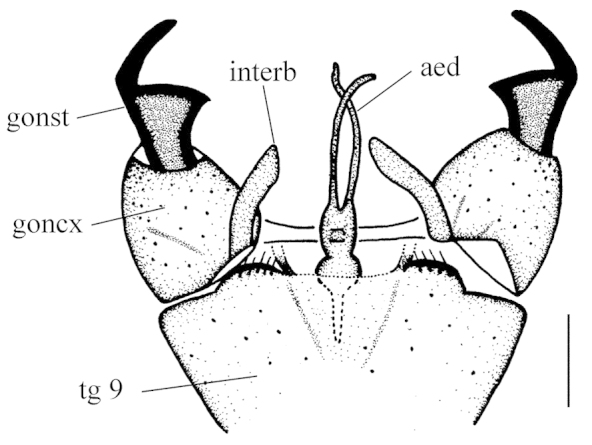
Toxorhina (Ceratocheilus) formosensis Alexander, 1928. Holotype. Male hypopygium, dorsal view. Scale bar: 0.2 mm.

Hypopygium (Fig. 2). Gonocoxite conical, relatively short and stout. Gonostylus stout and entirely blackened, outer lateral angle with a slender rod. Interbase compressed and slightly curved to aedeagus, tip blunt. Aedeagus with tip divergent, rods long filiform.

Female. Unknown.

##### Specimens examined.

**Holotype** male (USNM), China: Taiwan, Mt. Rantaizan (1829 m), 1927.VI.3, Syuti Issiki. (One wing and hypopygium are mounted on a similarly labeled microscope slide. Two fore legs and two mid legs are still attached to the dry mounted body, and two hind legs are absent.)

##### Distribution.

China (Taiwan).

##### Remarks.

For description and illustration of this species, also see Alexander (1928).

#### Toxorhina (Ceratocheilus) fuscolimbata

Taxon classificationAnimaliaDipteraLimoniidae

Alexander, 1967

Figs 3 , 4

Toxorhina (Ceratocheilus) fuscolimbata Alexander 1967: 185. Type locality: Hkayam Boum, Manipur, Assa, India.

##### Diagnosis.

Rostrum shorter than wing. Prescutum brownish yellow with three broad and nearly confluent brownish black stripes. Pleuron yellow with two black stripes. Wing tinged pale grey, black seams along cord and m-m and paler seam over base of CuA; R_2+3_ ending beyond end of basal section of R_4+5_, basal section of CuA_1_ beyond fork of M and at one-third of cell dm. Abdomen with sternites more or less bicolored. Male hypopygium with two pairs of gonostyl. Rods of aedeagus short.

##### Description.

Male. Body length 5.0–5.5 mm, wing length 5.5–6.0 mm, rostrum length 3.5 mm.

**Figure 3.**
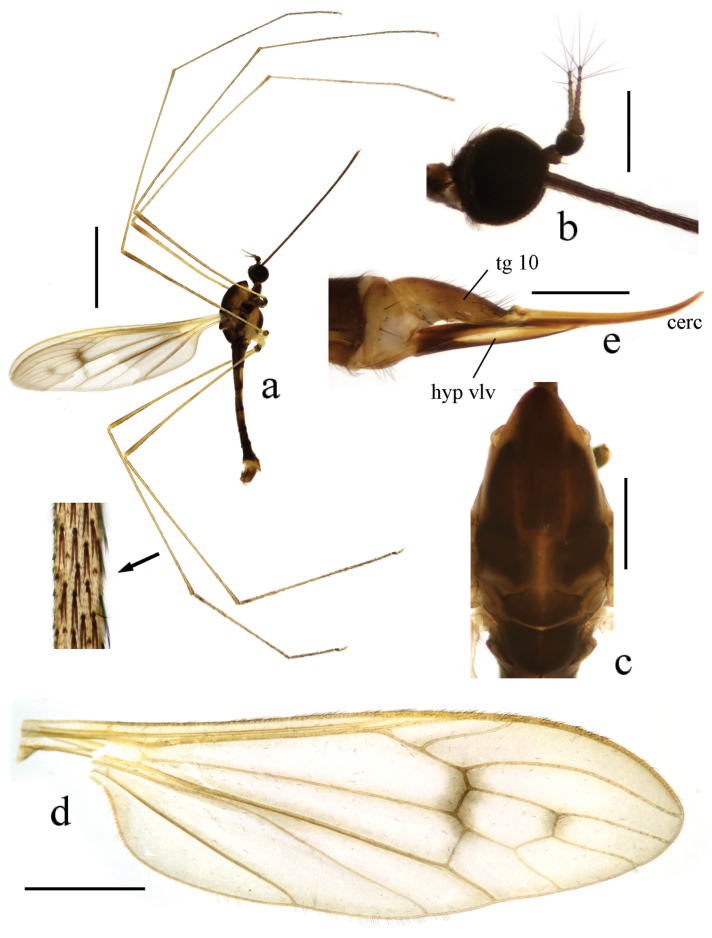
Toxorhina (Ceratocheilus) fuscolimbata Alexander, 1967. **a** Male habitus, lateral view **b** Head, lateral view **c** Thorax, dorsal view **d** Wing **e** Female hypopygium, lateral view. Scale bar: **a** = 2.0 mm; **b** = 0.5 mm; **c** = 0.5 mm; **d** = 1.0 mm; **e** = 0.3 mm.

Head (Fig. 3b). Brownish black. Hairs on head black. Antenna length 0.5–0.6 mm. Scape brownish black; pedicel black; flagellomeres brownish black. Pedicel enlarged and nearly globose. First flagellomere subconical; remaining flagellomeres cylindrical, each flagellomere longer and slenderer than previous one, terminal two flagellomeres longest with several long hairs. Rostrum brownish black with black hairs.

Thorax. Pronotum brownish black. Prescutum brownish yellow with three broad and nearly confluent brownish black stripes. Scutum brownish black with middle area paler, each lobe with a light yellow spot. Scutellum and mediotergite brownish black (Fig. 3c). Pleuron (Fig. 3a) yellow with two black stripes, upper one extending from cervical region to mediotergite, lower one extending from fore coxa to middle coxa. Hairs on thorax black. Fore coxa brownish black, middle and hind coxae pale yellow; trochanters brownish black; femora yellow to brownish yellow with tips darker; tibiae and tarsi brownish yellow. Hairs on legs black. Wing (Fig. 3d) tinged pale grey, black seams along cord and m-m and paler seam over base of CuA; veins pale brown, darker at CuA and A_2_ and in clouded areas. Venation: Sc_1_ ending a very short distance beyond origin of Rs, Sc_2_ a greater distance before origin of Rs; R_2+3_ ending beyond end of basal section of R_4+5_; basal section of CuA_1_ beyond fork of M and at one-third of cell dm; A_1_ curved suddenly at middle, basal half nearly straight. Haltere length 0.8–0.9 mm, white.

Abdomen (Fig. 3a). Tergites dark brown. Sternites brownish yellow with caudal 1/3 to 1/2 dark brown, sometomes uniformly dark brown. Segments six to eight uniformly dark brown to brownish black. Segment nine yellow to brownish yellow. Hairs on abdomen brownish black.

**Figure 4.**
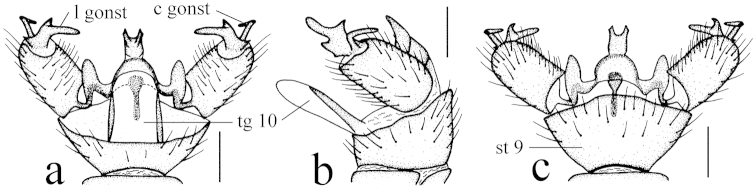
Toxorhina (Ceratocheilus) fuscolimbata Alexander, 1967. **a** Male hypopygium, dorsal view **b** Male hypopygium, lateral view **c** Male hypopygium, ventral view. Scale bar: **a–c** = 0.2 mm.

Hypopygium (Fig. 4). Generally dark brown. Gonocoxite cylindrical. Clasper of gonostylus slender and rod-shaped, curved ventrally and inwards, tip acute. Lobe of gonostylus with two stout spines near base. Interbase oval. Aedeagus with tip divergent, rods short.

Female. Body length 6.0–7.0 mm, wing length 5.5–6.0 mm, rostrum length 3.5–3.8 mm. Similar to male. But antenna obviously longer, each flagellomere subequal. Tenth tergite pale brown. Cercus dark brownish yellow. Hypogynial valve brownish yellow with base brownish black. Tip of hypogynial valve reaching at 2/5 of cercus (Fig. 4e).

##### Specimens examined.

2 males 2 females (CAU), China: Guangxi, Baise, Mt. Cenwanglao (24°29'12"N, 106°20'55"E, 1300 m), 2013.VII.29, Yuting Dai & Mengchao Tan. 4 males (CAU), China: Yunnan, Gongshan, Dulongjiang (27°40'32"N, 98°20'3"E, 1542 m), 2013.VII.1, Wei Zhang. 1 male (CAU), China: Xizang, Yigong (2300 m), 1978.VII.29, Fasheng Li.

##### Distribution.

China (Guangxi, Yunnan, Xizang); India.

##### Remarks.

This species is recorded from China for the first time. For description and illustration of this species, also see Alexander (1967, 1970).

#### Toxorhina (Ceratocheilus) huanglica sp. n.

Taxon classificationAnimaliaDipteraLimoniidae

http://zoobank.org/F286A8EA-9BD6-4632-AADB-82D42E460502

Figs 5 , 6

##### Diagnosis.

Rostrum shorter than wing. Prescutum brownish yellow with three broad dark brown stripes. Pleuron dark yellow with two black stripes. Wing tinged pale brown; R_2+3_ ending at or slightly beyond end of basal section of R_4+5_, basal section of CuA_1_ at or slightly before fork of M. Abdomen bicolored. Gonostylus with a stout spine near middle. Rods of aedeagus relatively short.

##### Description.

Male. Body length 5.0–5.5 mm, wing length 4.5–5.0 mm, rostrum length 3.5 mm.

**Figure 5.**
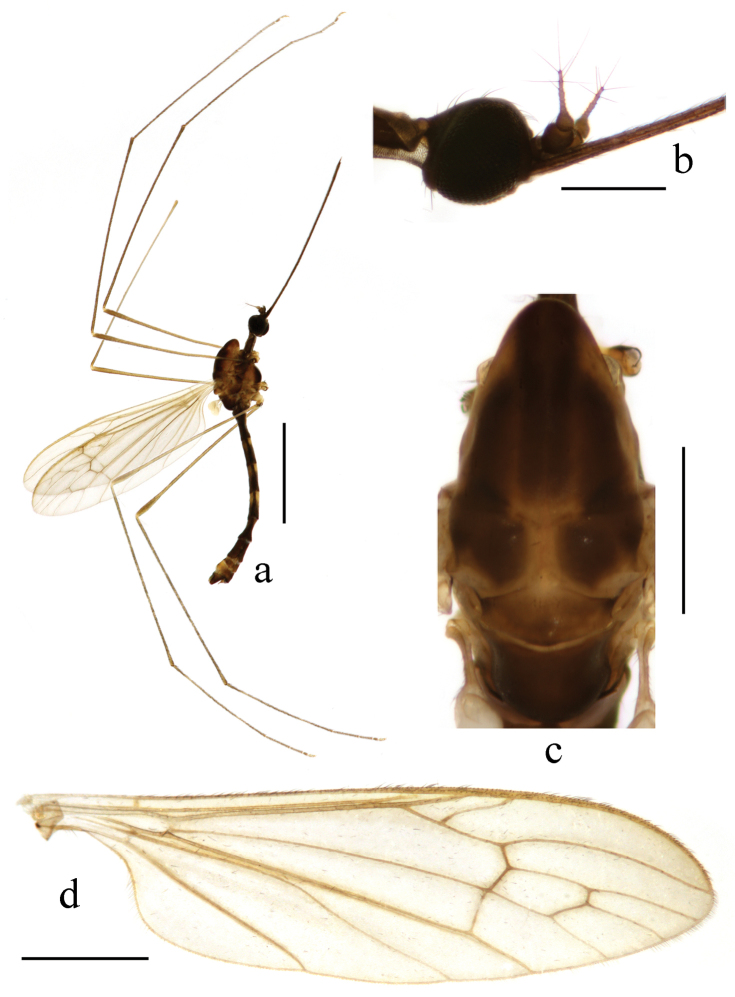
Toxorhina (Ceratocheilus) huanglica sp. n. **a** Male habitus, lateral view **b** Head, lateral view **c** Thorax, dorsal view **d** Wing. Scale bar: **a** = 2.0 mm; **b** = 0.5 mm; **c** = 0.5 mm; **d** = 1.0 mm.

Head (Fig. 5b). Brownish black. Hairs on head black. Antenna length 0.6–0.7 mm. Scape and pedicel dark brown; flagellomeres brown. Pedicel enlarged and nearly globose. First flagellomere subconical; remaining flagellomeres cylindrical, each flagellomere longer and slenderer than previous one, terminal two flagellomeres longest with several long hairs. Rostrum brownish black with black hairs.

Thorax. Pronotum dark brown. Prescutum brownish yellow with three broad dark brown stripes. Scutum dark brown with middle area paler, each lobe with a light yellow spot. Scutellum brown. Mediotergite dark brown to brownish black (Fig. 5c). Pleuron (Fig. 5a) dark yellow with two black stripes, upper one extending from cervical region to mediotergite, lower one extending from fore coxa to middle coxa. Hairs on thorax brownish black. Fore and middle coxa brownish yellow, hind coxae pale yellow; trochanters brownish black; femora brownish yellow with tips darker; tibiae and tarsi dark brown. Hairs on legs black. Wing (Fig. 5d) tinged pale brown; veins pale brown, darker at cord, m-m and basal section of M_3_. Venation: Sc_1_ a short distance beyond origin of Rs, Sc_2_ a slightly greater distance before origin of Rs; R_2+3_ ending at or slightly beyond end of basal section of R_4+5_; basal section of CuA_1_ at or slightly before fork of M; A_1_ curved suddenly at middle, basal half nearly straight. Haltere length 0.7–0.8 mm, pale yellow.

Abdomen (Fig. 5a). Tergites and sternites yellow with caudal halves brownish black. Segments six to eight uniformly brownish black. Tergite nine yellow. Sternite nine brownish yellow to brown. Hairs on abdomen brownish black.

**Figure 6.**
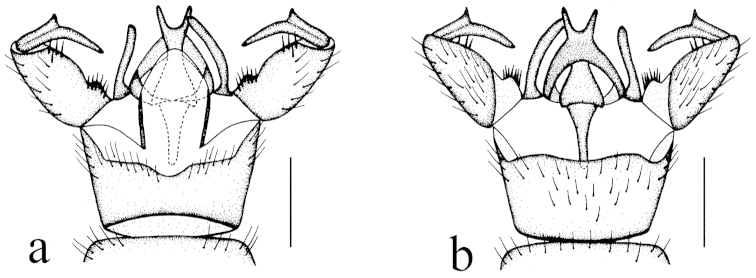
Toxorhina (Ceratocheilus) huanglica sp. n. **a** Male hypopygium, dorsal view **b** Male hypopygium, ventral view. Scale bar: **a–b** = 0.2 mm.

Hypopygium (Fig. 6). Generally dark brown. Gonocoxite conical, dorsal face with several setae and a blunt lobe near base, this lobe with numerous setae. Gonostylus with a stout spine near middle, basal half stout with a longitudinal groove. Interbase rod-shaped. Aedeagus with tip divergent, rods relatively short.

Female. Unknown.

##### Type material.

**Holotype** male (CAU), China: Yunnan, Lvchun, Mt. Huanglian, Qimaba (22°48'36"N, 102°14'24"E, 1012 m), 2013.VI.11, Jinying Yang (light trap). **Paratypes:** 4 males (CAU), same data as holotype.

##### Distribution.

China (Yunnan).

##### Etymology.

The species is named after the type locality Mt. Huanglian.

##### Remarks.

This new species is somewhat similar to Toxorhina (Ceratocheilus) fuscolimbata in having the similar body color, but it can be easily distinguished from the latter by the wing without conspicuous black seams and with basal section of CuA_1_ at or slightly before fork of M (Fig. 5d), the male hypopygium with one pair of gonostyl, the gonocoxite with a blunt lobe near base, and the interbase rod-shaped (Fig. 6). In Toxorhina (Ceratocheilus) fuscolimbata, the wing has the black seams along several veins, the basal section of CuA_1_ is beyond fork of M (Fig. 3d), the male hypopygium has two pairs of gonostyl, the gonocoxite has no lobe near base, and the interbase is oval (Fig. 4).

#### Toxorhina (Ceratocheilus) omnifusca sp. n.

Taxon classificationAnimaliaDipteraLimoniidae

http://zoobank.org/F9AB2E53-3F90-49B6-BCFC-E1DC0069B28F

Figs 7 , 8

##### Diagnosis.

Rostrum shorter than wing. Prescutum dark brown. Pleuron dark brown with central region slightly darker. Wing tinged pale brown; R_2+3_ ending at or slightly beyond end of basal section of R_4+5_, basal section of CuA_1_ at or slightly before fork of M. Abdomen dark brown with with segments six to eight slightly darker. Male hypopygium with two pairs of gonostyl. Rods of aedeagus relatively short.

##### Description.

Male. Body length 6.0 mm, wing length 5.5 mm, rostrum length 3.5 mm.

**Figure 7.**
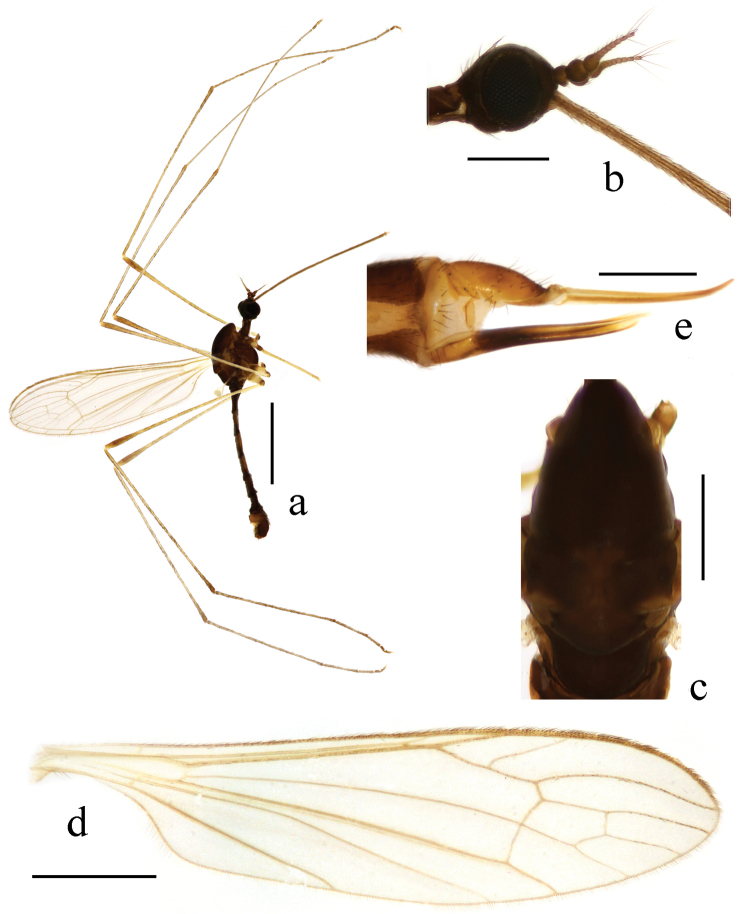
Toxorhina (Ceratocheilus) omnifusca sp. n. **a** Male habitus, lateral view **b** Head, lateral view **c** Thorax, dorsal view **d** Wing **e** Female hypopygium, lateral view. Scale bar: **a** = 2.0 mm; **b** = 0.5 mm; **c** = 0.5 mm; **d** = 1.0 mm; **e** = 0.4 mm.

Head (Fig. 7b). Brownish black. Hairs on head brownish black. Antenna length 0.6–0.7 mm. Scape and pedicel dark brown; flagellomeres brown to brownish yellow. Pedicel enlarged and nearly globose. First flagellomere subconical; remaining flagellomeres cylindrical, each flagellomere longer and slenderer than previous one, terminal two flagellomeres longest with several long hairs. Rostrum brownish yellow with brown hairs.

Thorax. Generally dark brown. Pronotum and prescutum dark brown. Scutum dark brown with middle area paler, each lobe with a paler spot. Scutellum brown with borders darker. Mediotergite dark brown (Fig. 7c). Pleuron (Fig. 7a) dark brown with central region slightly darker. Hairs on thorax dark brown. Coxae yellow to brownish yellow; trochanters brown; femora brownish yellow with bases yellow and tips pale brown; tibiae brownish yellow with tips pale brown; tarsi brownish yellow. Hairs on legs brown. Wing (Fig. 7d) tinged pale brownish yellow; veins brownish yellow. Venation: Sc_1_ just brfore origin of Rs, Sc_2_ near its tip; R_2+3_ ending beyond end of basal section of R_4+5_; basal section of CuA_1_ at fork of M; A_1_ curved suddenly at middle, basal half straight. Haltere length 0.8 mm, white.

Abdomen (Fig. 7a). Dark brown with segments six to eight slightly darker. Hairs on abdomen dark brown.

**Figure 8.**
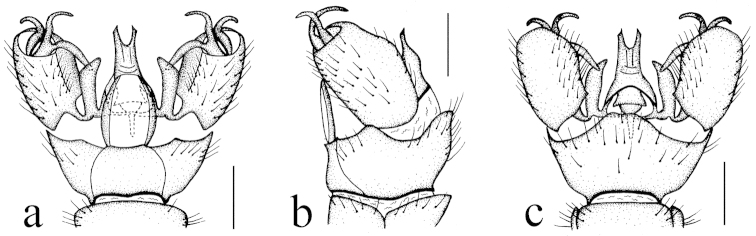
Toxorhina (Ceratocheilus) omnifusca sp. n. **a** Male hypopygium, dorsal view **b** Male hypopygium, lateral view **c** Male hypopygium, ventral view. Scale bar: **a–c** = 0.2 mm.

Hypopygium (Fig. 8). Generally brown to dark brown. Gonocoxite cylindrical. Clasper of gonostylus slender and rod-shaped, curved dorsally and outwards, tip acute. Lobe of gonostylus divided into two branches: outer one slender, curved ventrally and inwards; inner one relatively stout, strongly curved inwards. Interbase nearly oval, tip curved inwards. Aedeagus with tip divergent, rods relatively short.

Female. Body length 8.5 mm, wing length 6.5 mm, rostrum length 4.0 mm. Similar to male. Abdomen uniformly brown except hypopygium. Tenth tergite dark yellow to brownish yellow. Cercus brownish yellow with base paler. Hypogynial valve brownish yellow to brown with base yellow and middle area darker. Tip of hypogynial valve reaching just beyond middle of cercus (Fig. 7e).

##### Type material.

**Holotype** male (CAU), China: Sichuan, Pingwu, Laohegou (32°18'33"N, 104°42'45"E, 1100 m), 2012.V.22, Yang Li & Sipei Liu (light trap). **Paratype:** 1 female (CAU), same data as holotype.

##### Distribution.

China (Sichuan).

##### Etymology.

The specific epithet is an adjective and refers to the almost uniformly dark brown body (from Latin *omnifuscus* = *omni*- (adj., meaning “all”) + *fuscus* (adj., meaning “brown”)).

##### Remarks.

This new species is somewhat similar to Toxorhina (Ceratocheilus) tinctipennis in having the similar body color and venation of wing, but it can be easily distinguished from the latter by the male hypopygium with two pairs of gonostyl, the gonocoxite without lobe near base, and the interbase nearly oval (Fig. 8). In Toxorhina (Ceratocheilus) tinctipennis, the male hypopygium has one pair of gonostyl, the gonocoxite has a blunt lobe near base, and the interbase is rod-shaped (Fig. 12).

#### Toxorhina (Ceratocheilus) taiwanicola

Taxon classificationAnimaliaDipteraLimoniidae

(Alexander, 1923)

Figs 9 , 10

Ceratocheilus taiwanicola Alexander 1923: 475. Type locality: Tattaka, China (Taiwan).

##### Diagnosis.

Rostrum shorter than wing. Prescutum brownish yellow with three broad dark brown stripes. Pleuron dark brown with ventral region paler. Wing tinged pale grey; R_2+3_ ending before end of basal section of R_4+5_, basal section of CuA_1_ before fork of M. Abdomen dark brown. Gonostylus with a relatively small spine near middle. Rods of aedeagus relatively short.

##### Description.

Male. Body length 4.3–4.6 mm, wing length 4.5–5.4 mm, rostrum length 3.5 mm.

**Figure 9.**
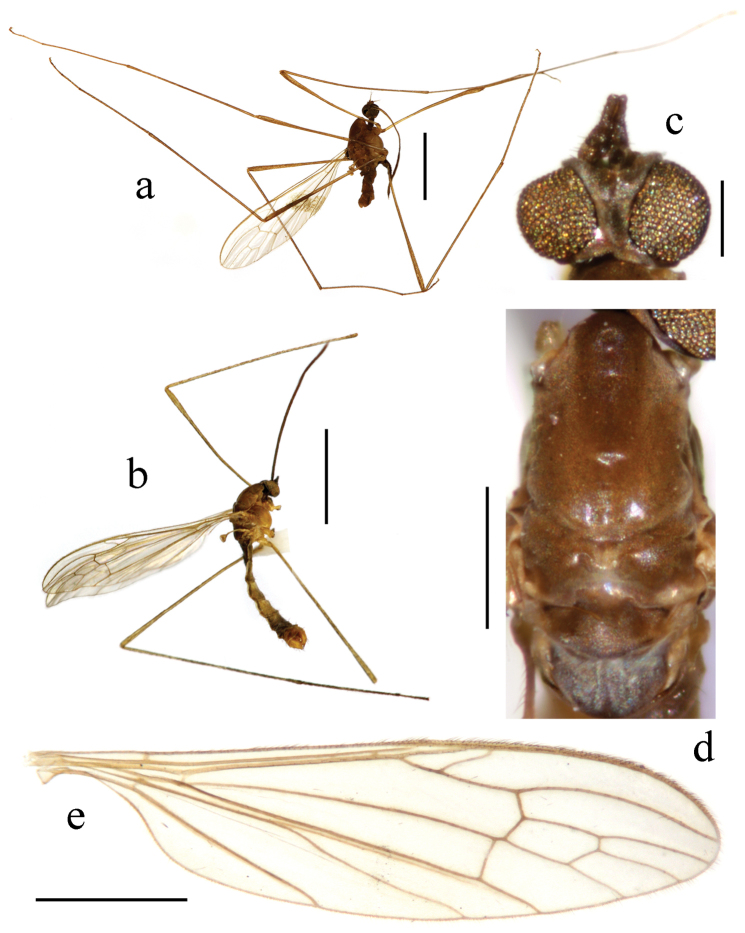
Toxorhina (Ceratocheilus) taiwanicola (Alexander, 1923). **a** Male habitus (holotype), lateral view **b** Male habitus, lateral view **c** Head, dorsal view **d** Thorax, dorsal view **e** Wing. Scale bar: **a** = 2.0 mm; **b** = 2.0 mm; **c** = 0.2 mm; **d** = 0.5 mm; **e** = 1.0 mm.

Head (Fig. 9c). Dark brown. Hairs on head brownish black. Antenna length 0.4 mm, dark brown. Pedicel enlarged and nearly globose. First flagellomere subconical; remaining flagellomeres cylindrical, each flagellomere longer and slenderer than previous one, terminal two flagellomeres longest with several long hairs. Rostrum brownish black with black hairs.

Thorax. Generally brownish yellow to brown. Pronotum dark brown. Prescutum brownish yellow with three broad dark brown stripes. Scutum, scutellum and mediotergite dark brown (Fig. 9d). Pleuron (Fig. 9a–b) dark brown with ventral region paler. Hairs on thorax dark brown. Coxa and trochanters pale brownish yellow; remainder of legs dark brown. Hairs on legs brownish black. Wing (Fig. 9e) tinged pale grey; veins pale brown. Venation: Sc_1_ ending a short distance beyond origin of Rs, Sc_2_ a slightly greater distance before origin of Rs; R_2+3_ ending before end of basal section of R_4+5_; basal section of CuA_1_ before fork of M; A_1_ curved suddenly at middle, basal half straight. Haltere length 0.7–0.8 mm, pale brownish yellow.

Abdomen (Fig. 9a–b). Dark brown, only hypopygium paler. Hairs on abdomen dark brown.

**Figure 10.**
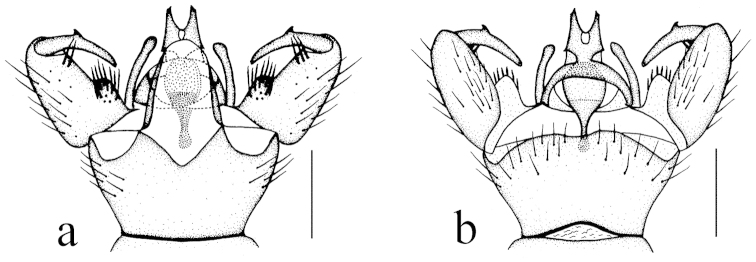
Toxorhina (Ceratocheilus) taiwanicola (Alexander, 1923). **a** Male hypopygium, dorsal view **b** Male hypopygium, ventral view. Scale bar: **a–b** = 0.2 mm.

Hypopygium (Fig. 10). Generally brownish yellow. Gonocoxite conical, dorsal face with two or three setae near tip and a blunt lobe near base, this lobe with numerous setae. Gonostylus with a relatively small spine near middle. Interbase rod-shaped, tip slightly enlarged. Aedeagus with tip divergent, rods relatively short.

Female. Unknown.

##### Specimens examined.

**Holotype** male (USNM), China: Taiwan, Tattaka (2250 m), 1921.VIII.18, Teiso Esaki. (One wing and hypopygium are mounted on a similarly labeled microscope slide. All six legs are still attached to the dry mounted body.) **Other material:** 1 male (CAU), China: Hainan, Ledong, Jianfengling, Mingfenggu (18°42'32"N, 108°49'41"E, 800 m), 2007.X.25, Xingyue Liu.

##### Distribution.

China (Jiangxi, Taiwan, Hainan).

##### Remarks.

Male hypopygium of this species is described in detail and illustrated for the first time. For description of this species, also see Alexander (1923). Alexander (1923) described the basal section of CuA_1_ as “about one-fifth its length beyond the fork of M”, whereas it is before the fork of M in both the holotype (Fig. 9a) and the specimen we examined (Fig. 9e).

#### Toxorhina (Ceratocheilus) tinctipennis

Taxon classificationAnimaliaDipteraLimoniidae

(Alexander, 1930)

Figs 11 , 12

Ceratocheilus tinctipennis Alexander 1930: 75. Type locality: Mt. Ali (Arishan), China (Taiwan).

##### Diagnosis.

Rostrum shorter than wing. Prescutum brownish black. Pleuron brownish black. Wing with a strong brown suffusion; R_2+3_ ending beyond or close to end of basal section of R_4+5_, basal section of CuA_1_ before or close to fork of M. Abdomen dark brown with segments six to eight darker. Gonostylus with a very small spine near middle. Rods of aedeagus short.

##### Description.

Male. Body length 5.0–6.0 mm, wing length 4.5–5.0 mm, rostrum length 3.0–3.5 mm.

**Figure 11.**
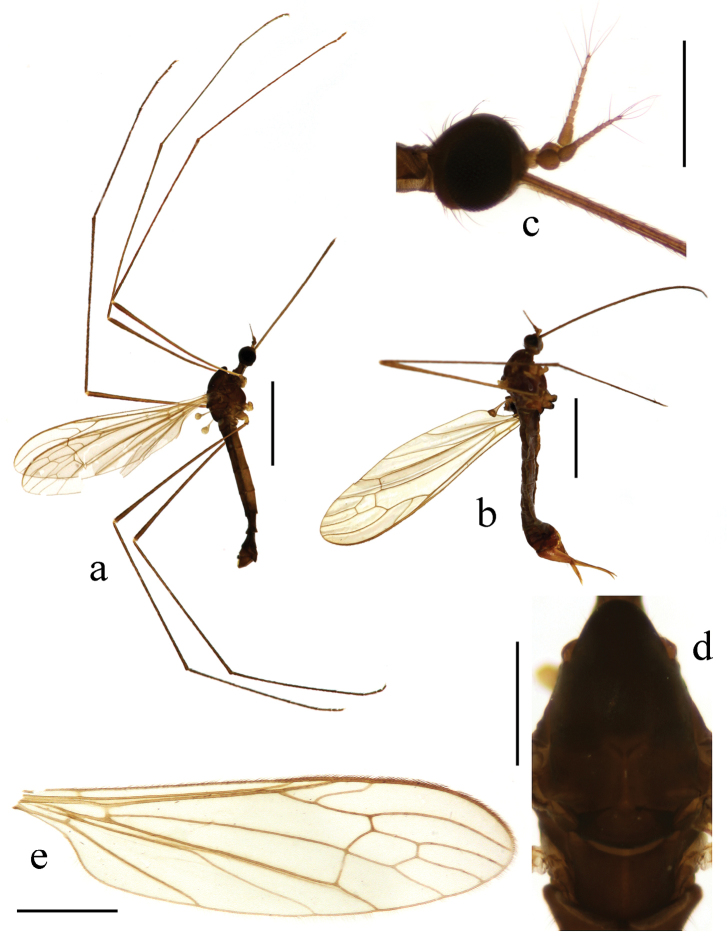
Toxorhina (Ceratocheilus) tinctipennis (Alexander, 1930). **a** Male habitus, lateral view **b** Female habitus (holotype), lateral view **c** Head, dorsal view **d** Thorax, dorsal view **e** Wing. Scale bar: **a** = 2.0 mm; b = 2.0 mm; **c** = 0.5 mm; **d** = 0.5 mm; **e** = 1.0 mm.

Head (Fig. 11c). Dark brown. Hairs on head dark brown. Antenna length 0.6–0.7 mm. Scape brown; pedicel and flagellomeres dark brown. Pedicel enlarged and nearly globose. First flagellomere subconical; remaining flagellomeres cylindrical, each flagellomere longer and slenderer than previous one, terminal two flagellomeres longest with several long hairs. Rostrum dark brown to brownish black with dark brown hairs.

Thorax. Generally dark brown to brownish black. Pronotum dark brown. Prescutum brownish black. Scutum dark brown with middle area slightly paler. Scutellum dark brown with borders brownish black to black. Mediotergite dark brown (Fig. 11d). Pleuron (Fig. 11a–b) brownish black. Hairs on thorax dark brown. Coxae brownish yellow; trochanters yellow; femora dark brown with bases paler; tibiae and tarsi dark brown to brownish black. Hairs on legs dark brown. Wing (Fig. 11e) with a strong brown suffusion; veins dark brown. Venation: Sc_1_ ending a short distance beyond origin of Rs, Sc_2_ same or a slightly greater distance before origin of Rs; R_2+3_ ending beyond or close to end of basal section of R_4+5_; basal section of CuA_1_ before or close to fork of M; A_1_ curved suddenly at middle, basal half straight. Haltere length 0.7–0.8 mm, brownish yellow to pale brown.

Abdomen (Fig. 11a–b). Generally dark brown. Segments six to eight darker. Hairs on abdomen dark brown.

**Figure 12.**
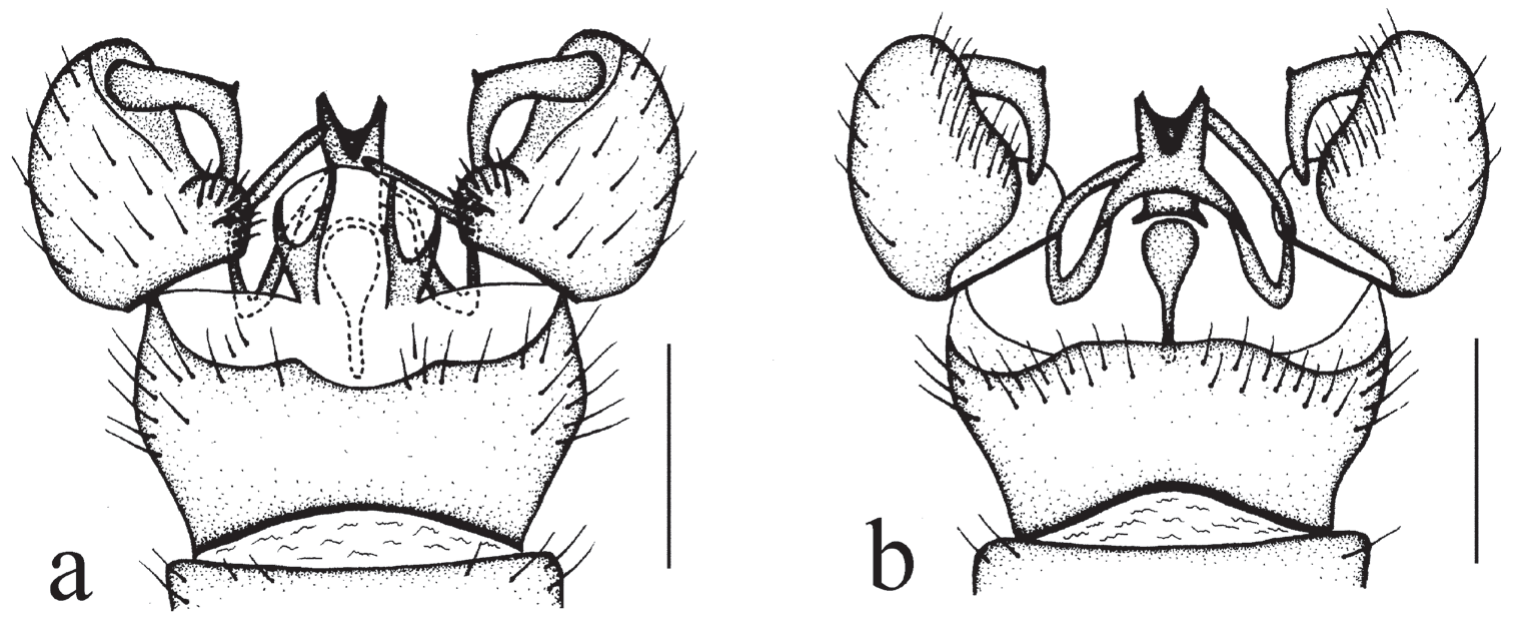
Toxorhina (Ceratocheilus) tinctipennis (Alexander, 1930). **a** Male hypopygium, dorsal view **b** Male hypopygium, ventral view. Scale bar: **a–b** = 0.2 mm.

Hypopygium (Fig. 12). Generally dark brown. Gonocoxite conical, short and stout, dorsal face with a blunt lobe near base, this lobe with numerous setae. Gonostylus with a very small spine near middle. Interbase rod-shaped. Aedeagus with tip divergent, rodS short.

Female (Fig. 11b). Body length 7.0–7.5 mm, wing length 6.0–6.5 mm, rostrum length 4.0–4.5 mm. Similar to male. But abdomen except hypopygium uniformly dark brown. Tenth tergite brownish yellow. Cercus and hypogynial dark brownish yellow. Tip of hypogynial valve reaching just beyond middle of cercus.

##### Specimens examined.

**Holotype** female (USNM), China: Taiwan, Jiayi, Mt. Ali (1981–2438 m), 1929.VII.7, Syuti Issiki. (One wing and one fore leg are mounted on a similarly labeled microscope slide. Only one mid leg is still attached to the dry mounted body, and the remaining four legs are absent.) **Other materials:** 8 males 1 female (CAU), China: Taiwan, Jiayi, Mt. Ali (23°26'24"N, 120°46'48"E, 1100 m), 2012.VI.7, Lihua Wang.

##### Distribution.

China (Taiwan).

##### Remarks.

Male of this species is described and illustrated for the first time. For description and illustration of this species, also see Alexander (1930).

#### Toxorhina (Ceratocheilus) univirgata sp. n.

Taxon classificationAnimaliaDipteraLimoniidae

http://zoobank.org/160ED401-82B6-4CA4-ABCF-348F53F81562

Figs 13 , 14

##### Diagnosis.

Prescutum brownish yellow with three broad brown stripes. Pleuron yellow with one dark brown stripe. Wing tinged pale grey; R_2+3_ ending beyond end of basal section of R_4+5_, basal section of CuA_1_ slightly before fork of M. Sternites of abdomen brown with basal several segments paler. Gonostylus with base stout, middle with a stout and ventrally curved spine. Rods of aedeagus very long.

##### Description.

Male. Body length 5.5 mm, wing length 5.5 mm, rostrum broken with remaining part length 1.5 mm.

**Figure 13.**
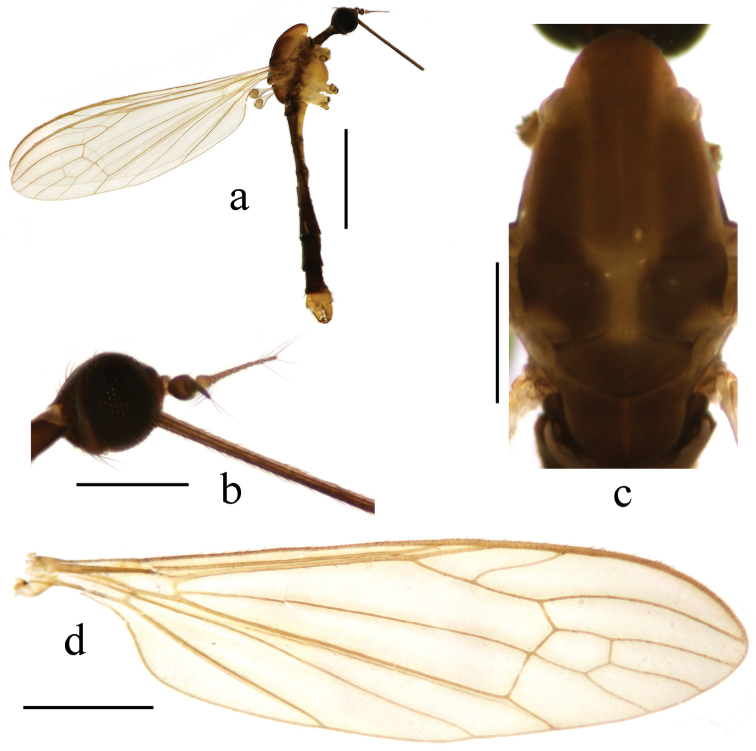
Toxorhina (Ceratocheilus) univirgata sp. n. **a** Male habitus, lateral view **b** Head, lateral view **c** Thorax, dorsal view **d** Wing. Scale bar: **a** = 2.0 mm; **b** = 0.5 mm; **c** = 0.5 mm; **d** = 1.0 mm.

Head (Fig. 13b). Dark brown to brownish black. Hairs on head dark brown. Antenna length 0.6 mm. Scape and pedicel dark brown; flagellomeres brown. Pedicel enlarged and nearly globose. First flagellomere subconical; remaining flagellomeres cylindrical, each flagellomere longer and slenderer than previous one, terminal two flagellomeres longest with several long hairs. Rostrum dark brown with dark brown hairs.

Thorax. Pronotum dark brown. Prescutum brownish yellow with three broad brown stripes. Scutum dark brown with middle area paler, each lobe with a light yellow spot. Scutellum and mediotergite dark brown (Fig. 13c). Pleuron (Fig. 13a) yellow with one dark brown stripe extending from cervical region to base of abdomen. Hairs on thorax dark brown. Coxae pale yellow; trochanters yellow with tips black; other parts missing. Wing (Fig. 13d) tinged pale grey; veins pale brown. Venation: Sc_1_ ending near middle of Rs, Sc_2_ before origin of Rs; R_2+3_ ending beyond end of basal section of R_4+5_; basal section of CuA_1_ slightly before fork of M; A_1_ curved relatively smoothly. Haltere length 0.8 mm, pale yellow with knob darker.

Abdomen (Fig. 13a). Tergites brownish black. Sternites brown with basal several segments paler. Segment five to eight black with segment eight paler. Segment nine yellow. Hairs on abdomen brownish black.

**Figure 14.**
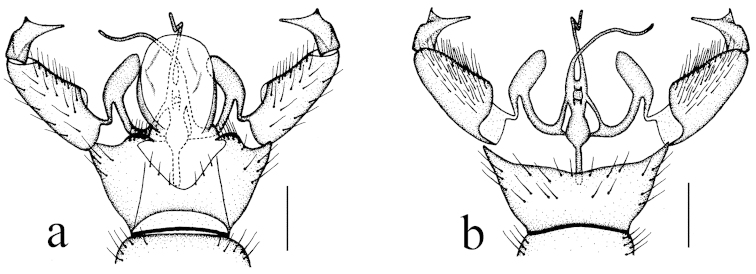
Toxorhina (Ceratocheilus) univirgata sp. n. **a** Male hypopygium, dorsal view **b** Male hypopygium, ventral view. Scale bar: **a–b** = 0.2 mm.

Hypopygium (Fig. 14). Generally yellow to pale brownish yellow. Gonocoxite conical. Gonostylus with base stout, middle with a stout and ventrally curved spine. Interbase nearly oval, tip blunt. Aedeagus with tip divergent, rods filiform and very long.

Female. Unknown.

##### Type material.

**Holotype** male (CAU), China: Yunnan, Lvchun, Mt. Huanglian (26°34'36"N, 113°28'12"E, 1542 m), 2013.VII.25, Mengchao Tan (light trap).

##### Distribution.

China (Yunnan).

##### Etymology.

The specific epithet is an adjective and refers to the dark brown stripe on pleuron (from Latin *univirgatus* = *uni*- (adj., meaning “single”) + *virgatus* (adj., meaning striped)).

##### Remarks.

This new species is somewhat similar to Ceratocheilus (Ceratocheilus) fulvicolor in having the similar male hypopygium and wing, but it can be easily distinguished from the latter by the prescutum (Fig. 13c) brownish yellow with three broad brown stripes, pleuron (Fig. 13a) yellow with one dark brown stripe, and abdomen with tergites brownish black (Fig. 13a). In Toxorhina (Ceratocheilus) fulvicolor, the prescutum is pale brownish yellow and irregularly variegated, the pleuron is pale yellow, and the tergites of the abdomen are brownish yellow with the posterior borders narrowly brown (Alexander 1967).

## Supplementary Material

XML Treatment for Toxorhina (Ceratocheilus) formosensis

XML Treatment for Toxorhina (Ceratocheilus) fuscolimbata

XML Treatment for Toxorhina (Ceratocheilus) huanglica

XML Treatment for Toxorhina (Ceratocheilus) omnifusca

XML Treatment for Toxorhina (Ceratocheilus) taiwanicola

XML Treatment for Toxorhina (Ceratocheilus) tinctipennis

XML Treatment for Toxorhina (Ceratocheilus) univirgata
